# SMG: self-supervised masked graph learning for cancer gene identification

**DOI:** 10.1093/bib/bbad406

**Published:** 2023-11-08

**Authors:** Yan Cui, Zhikang Wang, Xiaoyu Wang, Yiwen Zhang, Ying Zhang, Tong Pan, Zhe Zhang, Shanshan Li, Yuming Guo, Tatsuya Akutsu, Jiangning Song

**Affiliations:** Bioinformatics Center, Institute for Chemical Research, Kyoto University, Kyoto 611-0011, Japan; Monash Biomedicine Discovery Institute and Department of Biochemistry and Molecular Biology, Monash University, Melbourne, VIC 3800, Australia; Monash Biomedicine Discovery Institute and Department of Biochemistry and Molecular Biology, Monash University, Melbourne, VIC 3800, Australia; School of Public Health and Preventive Medicine, Monash University, Melbourne, VIC 3004, Australia; School of Computer Science and Engineering, Nanjing University of Science and Technology, 200 Xiaolingwei, Nanjing, 210094, China; Monash Biomedicine Discovery Institute and Department of Biochemistry and Molecular Biology, Monash University, Melbourne, VIC 3800, Australia; UniDT, Jing'an, Shanghai, China; School of Public Health and Preventive Medicine, Monash University, Melbourne, VIC 3004, Australia; School of Public Health and Preventive Medicine, Monash University, Melbourne, VIC 3004, Australia; Bioinformatics Center, Institute for Chemical Research, Kyoto University, Kyoto 611-0011, Japan; Monash Biomedicine Discovery Institute and Department of Biochemistry and Molecular Biology, Monash University, Melbourne, VIC 3800, Australia; Monash Data Futures Institute, Monash University, Melbourne, VIC 3800, Australia

**Keywords:** cancer genes, self-supervised learning, graph learning, representation learning, protein–protein interaction network

## Abstract

Cancer genomics is dedicated to elucidating the genes and pathways that contribute to cancer progression and development. Identifying cancer genes (CGs) associated with the initiation and progression of cancer is critical for characterization of molecular-level mechanism in cancer research. In recent years, the growing availability of high-throughput molecular data and advancements in deep learning technologies has enabled the modelling of complex interactions and topological information within genomic data. Nevertheless, because of the limited labelled data, pinpointing CGs from a multitude of potential mutations remains an exceptionally challenging task. To address this, we propose a novel deep learning framework, termed self-supervised masked graph learning (SMG), which comprises SMG reconstruction (pretext task) and task-specific fine-tuning (downstream task). In the pretext task, the nodes of multi-omic featured protein–protein interaction (PPI) networks are randomly substituted with a defined mask token. The PPI networks are then reconstructed using the graph neural network (GNN)-based autoencoder, which explores the node correlations in a self-prediction manner. In the downstream tasks, the pre-trained GNN encoder embeds the input networks into feature graphs, whereas a task-specific layer proceeds with the final prediction. To assess the performance of the proposed SMG method, benchmarking experiments are performed on three node-level tasks (identification of CGs, essential genes and healthy driver genes) and one graph-level task (identification of disease subnetwork) across eight PPI networks. Benchmarking experiments and performance comparison with existing state-of-the-art methods demonstrate the superiority of SMG on multi-omic feature engineering.

## INTRODUCTION

Cancer genomics endeavours to elucidate the genetic and non-genetic factors that contribute to the evolution of tumour cells, with the primary goal of identifying cancer genes (CGs). These CGs are responsible for driving the initiation and progression of cancer and as such, are critical focus of cancer genomics studies [1–4]. Over the past decade, numerous large-scale cancer genomic projects have sprung up, leading to the generation and accumulation of genomic, epigenomic, transcriptomic and proteomic data from thousands of cancer patients, e.g. The Cancer Genome Atlas (TCGA) [5], the International Cancer Genome Consortium [6] and the Catalogue Of Somatic Mutations in Cancer [7]. Such high-throughput genomic data have significantly facilitated the research efforts for CG identification.

In recent years, as artificial intelligence and big data technologies have advanced, an increasing number of studies have focused on identifying CGs using data-driven computational approaches. For instance, based on the frequency hypothesis [8, 9] that CGs undergo mutations more frequently than expected in contrast to the background mutation rate (BMR) estimated from samples of a given cancer type, the MutSig [10] and MuSic [11] approaches were developed. Despite the progress, it is still challenging to estimate BMR properly—a low BMR may result in many false positives, whereas a high BMR may miss the CGs that are mutated with lower frequencies.

Moreover, many existing machine learning and deep learning methods attempted to directly extract the mutational patterns related to CGs from the genomic data by extensively exploring the functional impact of single-nucleotide variants on protein function [12–14] and functional genomics features (e.g. transcriptomic, epigenomics and proteomics) [15–17]. However, because of the complexities and heterogeneities of cancers, these methods often fail to achieve satisfactory performance in CG identification and discovery. Recent studies have shown that cancer often results from changes in intricate biological processes that interact within complex networks rather than being caused by the alteration of a single gene or protein [18]. In addition, the abnormalities in a specific gene or protein may propagate through its connections within the molecular network, leading to widespread effects. Therefore, protein–protein interaction (PPI)-based approaches [19–21] have garnered increased interests in recent years. In this regard, HotNet2 [19] is the first method that mapped genomic features onto the PPI networks for CG identification. It utilized a directed heat diffusion model to simultaneously assess the significance of mutations in individual genes and the local topology of interactions amongst the encoded proteins, offering the advantage of overcoming the limitations of pathway-based enrichment statistics. To improve the dependability of the featured PPI networks, some methods introduced other types of data, including gene expression profiles [20, 22–24], subcellular location [25] and transcriptional regulation [26]. EMOGI [20] innovatively constructed the graph by connecting the multi-omic data with the PPI networks. However, its exploration of the interaction and features of the unlabelled nodes was still insufficient and as a consequence, it has limited model performance and generalizability for CG identification. To tackle this problem, MTGCN [22] introduced multitask learning to classify nodes and predict link association simultaneously. In this way, the trained model can generate better node-level representations. Nevertheless, the improvement of MTGCN over EMOGI [20] was quite marginal, presumably because the auxiliary topological correlation learning task cannot provide sufficient node-level features during model training. In summary, the progress of CG identification is still considerably impeded by the scarcity of labelled data and as such, there is a large room for further improvement.

Self-supervised learning (SSL) techniques have recently demonstrated tremendous potential across many areas, such as computer vision [27, 28] and natural language processing tasks [29]. This machine learning approach utilizes unlabelled data to generate meaningful and functional representations of the input data. Current SSL algorithms can be categorized into contrastive-based SSL and generative-based SSL. Contrastive-based SSL often depends on structural data augmentation and intricate model training, considerably limiting its scalability to complex data formats. Generative-based SSL addresses the above issues by circumventing data augmentation with data self-reconstruction, making generative-based SSL easily extended to any kind of input. However, generative-based SSL has not been explored yet for CG identification to date.

This paper introduces a novel computational approach, termed self-supervised masked graph (SMG), which is developed based on SMG learning to address the scarcity of labelled data for CG identification. First, we adopt the strategy of EMOGI [20] to construct the multi-omic featured PPI networks. In the subsequent SMG reconstruction phase, we randomly replace some nodes with a predefined mask token (MASK) and utilize a graph neural network (GNN) autoencoder (AE) to reconstruct the masked nodes by referring to the neighbour information. In this way, we effectively capture the intricate interaction relationships between nodes whilst preserving the topological information. In the task-specific fine-tuning phase, we leverage the pre-trained GNN encoder to embed the PPI networks into the feature graphs and adopt a task-specific layer to make the prediction. We comprehensively compare our proposed SMG method with other state-of-the-art methods on three node-level tasks [e.g. CG, essential gene (EG) and healthy driver (HD) gene identification] and one graph-level task (disease subnetwork identification). The outstanding performance highlights the superiority of the proposed SMG method in dealing with the scarcity of labelled data. Meanwhile, SMG is also capable of identifying a number of putative CGs that have recently been experimentally discovered, suggesting its significant potential and scalability in unlocking new cancer-associated marker genes from the PPI data.

## MATERIALS AND METHODS

**Figure 1:**
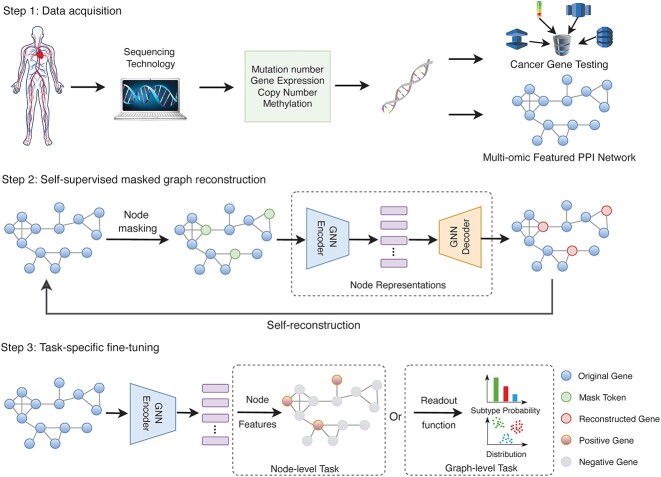
Overview of the proposed SMG learning. There exist three steps including data acquisition, SMG reconstruction and task-specific fine-tuning. In step 3, both node-level and graph-level tasks can be implemented based on the pre-trained GNN encoder.

This section briefly introduces the computational framework of SMG. As depicted in Figure 1, its framework comprises the pretext and downstream tasks, termed SMG reconstruction and task-specific fine-tuning, respectively. In the pretext phase, the GNN encoder and decoder jointly reconstruct the masked multi-omic featured PPI network. In the downstream phase, the pre-trained GNN encoder is applied to both the node-level and graph-level tasks.

### Data acquisition

In this study, we collected the gene mutation, copy number, DNA methylation and gene expression data of 29 446 samples covering 16 cancer types from the TCGA data sets [5]. We analyzed cancer types with DNA methylation data in both tumour and normal tissues and pre-processed batch effect-corrected gene expression data in accordance with the pre-processing procedures described in EMOGI [20].

We collected the PPI Networks from multiple public databases including CPDB [30], STRING-db [31], IRefIndex [32], Multinet [33] and PCNet [34]. The noisy nodes with low-confidence interactions were discarded to reduce the deviations of the networks. Particularly, we merely reserved the PPIs of CPDB and STRING-db networks with the confidence scores of higher than 0.5 and 0.85, respectively. In terms of Multinet and the older version of IRefIndex (2015), the PPI data were collected from the Hotnet2 [19] GitHub repository. In terms of PCNet, we followed Huang *et al*.’s work [34] without any postprocessing. In addition, we also collected the latest versions of the CPDB (2021) [63] and IRefIndex (2022) [67] networks to evaluate the model performance and reliability. We integrated the different PPI data into the same graph structure format by converting different format gene names into the uniform symbol names and setting each individual gene as a graph node, and then constructing the edges between different nodes based on the protein interactions. As a consequence, the raw data of each PPI network were transformed into the uniform formatted graph data.

To assess the usability and generalization of the proposed SMG framework, we tested the pre-trained GNN encoder on four different downstream tasks, including CG identification, EG identification, HD gene identification and disease subnetwork identification. In terms of CGs, both positive and negative examples were collected. The positive labels were referred to the Network of Cancer Genes (NCG) [35] and DigSee [36], whereas the negatives were randomly sampled from the remaining unlabelled genes. In particular, the number of negative samples in the six old version PPI networks (CPDB, STRING-db, IRefIndex, Multinet and PCNet) was referred to the EMOGI work to ensure the fair benchmark comparison; whereas in the latest PPI networks (CPDB (2021) [30] and IRefIndex (2022) [32]), we set the positive/negative ratio as 1:3, which was close to the previous benchmarking setting. The unselected unlabelled nodes were regarded as the unknown nodes during the fine-tuning phase, which were not involved in the loss function calculation and model optimization, but as the intermediate nodes, could serve to pass the message between the labelled nodes. With respect to the EGs that play important roles in human metabolism and survival, we collected them by summarizing 16 human EG data sets from the DEG database [37]. In accordance with Hong *et al*.’s work [38], we designated the genes that were present in at least five data sets as the positive samples, whereas those not found in any data set were regarded as the negative ones. HD genes are responsible for promoting non-cancerous cell clones. Labelling HD genes is extremely challenging, resulting in a relatively small number of identified HD genes. Similar to the CG setting, in the HD gene identification task, the remaining nodes in the PPI networks participated the model training as the intermediate unlabelled nodes. Accordingly, we collected the HD genes by referring to NCG [35]. For the disease subnetwork detection task, we collected the disease subtype labels directly from TCGA [5].

### SMG reconstruction

The reconstruction of SMG s follows the principle of masked AEs [39] on natural images and GraphMAE [40] on graphs. The training technique essentially seeks to reconstruct the masked nodes by referring to the neighbourhood information. According to the step 2 shown in Figure 1, a predefined MASK replaces the node features and indicates the masking position in the graph. Afterwards, the GNN encoder generates the representations for each node, whereas the GNN decoder reconstructs the whole graph. This strategy resembles the AE, where the largest difference is reflected by the input data. AE implements the self-reconstruction with the original inputs; however, SMG conducts the reconstruction with the masked broken data. This distinction enables SMG to effectively learn stronger correlations amongst the nodes and generate more discriminative representations. In the following section, we describe the masking strategy and network structure in detail.

#### Masking strategy

Graphs are complex data that have both node features and topological information. There are two main strategies for node masking: (1) erase the whole node from both topological and feature aspects and (2) merely mask the node features but preserve the topological information. The first strategy is rough and simple; however, the topological information that can reflect the biological process is missing, thus violating the assumption of self-prediction, i.e. the mask should not change the input data property. Therefore, we adopt the second strategy in the pre-training phase. Given an input graph $G=\left(V,E\right)$, we randomly replace some nodes with the pre-defined MASK and obtain $\overset{\sim }{G}=\left(\overset{\sim }{V},E\right)$. Note that the MASK has no semantic meaning but only indicates the masking position.

#### GNN encoder and GNN decoder

Depending on the downstream task, we construct the GNN encoder and GNN decoder accordingly. The main feature of GNN is reflected by the message passing mechanism, i.e. the node representation is updated by aggregating the neighbour nodes’ information. Many GNN-variant architectures, such as Graph Convolution Network (GCN) [41], GAT [42], Graph Isomorphism Network (GIN) [43], have been developed in recent years, whose principles can be formulated as follows:


(1) \begin{equation*} {a}_v^k={\text{Aggregator}}^k\left({f}_u^{k-1};u\in N(v)\right), \end{equation*}

(2) \begin{equation*} {f}_v^k={\text{Combine}}^k\left({f}_v^{k-1},{a}_v^k\right), \end{equation*}

where ${f}_v^k$ represents the node ${v}^{{\prime}}$s features on the *k*th layer of GNN and $N(v)$ denotes the neighbour nodes of $v$, respectively. Each layer’s iteration includes the aggregating and combining operations, and the main difference between various GNN architectures lies in the two operations.

##### Node classification task

Because of the inherent inductive bias of GCN [41], which prioritizes the importance of the local information during the feature learning procedure, the GCN architecture has achieved excellent performance in node representation learning. In light of this, we choose the GCN to construct our encoder and decoder for the node classification tasks. The aggregation and combination functions of GCN can be integrated as follows:


(3) \begin{equation*} {f}_v^k=\text{ReLU}\left(W\cdot \text{avg}\left({f}_u^{k-1}\right),\forall u\in N(v)\cup v\right), \end{equation*}

After constructing the encoder and decoder with GCN, the residual link between the two nearest layers for gradient passaging is added. This can help relieve the over-smoothing for training deep GNNs and preserve the important topological features. Afterwards, the LayerNorm [44] is appended after the hidden layer. This normalization operation can ensure fast convergence during the pre-training phase and scalable adaptation to the downstream tasks.

##### Graph classification task

Although the inductive bias of GCN inherited from convolutional neural networks makes it suitable for node-level classification, its performance may substantially drop when being applied to the graph-level tasks. Therefore, for the graph classification task, we utilized the GIN, one of the most representative GNN models based on the Weisfeiler–Lehman graph isomorphism test, to construct the encoder and decoder structures. Compared with GCN, GIN pays more attention to the global contextual information and as such, it is more suitable for graph-level representations. The node feature updating process can be formulated as follows:


(4) \begin{equation*} {f}_v^k={\text{MLP}}^k\left(\Big(1+{\varepsilon}^k\right){f}_v^{k-1}+\sum_{u\in N(v)}{f}_u^{k-1}\Big), \end{equation*}

where $\text{MLP}$ is the multilayer perceptron and $\varepsilon$ is a predefined hyperparameter. Moreover, different from node-level classification tasks, graph-level tasks require one more readout function to generate the final representation of the entire graph. Here, we chose the average operation as our readout function and generated the graph feature vectors after each layer. In terms of the architecture of the GCN/GIN-based encoder and decoder, we adopted a symmetric framework, for which both the encoder and decoder comprised three hidden layers with 256 hidden dimensions.

### Loss function

#### Loss for reconstruction

In the SMG reconstruction phase, we utilized the scaled cosine error loss [40] for graph reconstruction. Specifically, the loss function can be formulated as follows:


(5) \begin{equation*} {L}_{\text{SCE}}=\frac{1}{\mid V\mid}\sum_{v_i\in V}\left(1-\frac{f_i^T{v}_i}{\left\Vert{f}_i^T\right\Vert \cdot \left\Vert{v}_i\right\Vert}\right), \end{equation*}

where ${f}_i$ and ${v}_i$ represent the reconstructed feature and the original feature of the *i*th node, respectively, whereas $\mid V\mid$ indicates the number of nodes in the graph.

#### Loss for task-specific fine-tuning

After the SMG reconstruction, we conducted task-specific fine-tuning. First, the pre-trained GNN encoder generated a high-dimensional feature vector for each node. For the node-level task, the feature vectors were simply fed into a learnable classification layer to project the representations from the feature space to the probability space. During this process, we optimized both the pre-trained GNN encoder and classification layer with the assistance of the weighted masked binary cross entropy loss (wmBCE). In contrast to the standard binary cross entropy loss, wmBCE only propagated a small portion of the label samples’ gradient for model training based on transductive learning setting, the loss function was reweighted with the positive–negative sample ratio to complement with the gradient propagating strategy. The masking and reweighting strategies jointly alleviated the class imbalance. Given the output probability $p$, the ground-truth label $y$, the positive–negative ratio $r$ and $\text{sigmoid}$ activate function $\sigma$, weBCE can be formulated as follows:


(6) \begin{equation*} {L}_{\text{wmBCE}}=-w\left[r\cdot y\cdot \log \right(\sigma \left(p\Big)\right)+\left(1-y\right)\cdot \log \left(1-\sigma (p)\right)\Big]. \end{equation*}

In terms of the graph-level task, we utilized a linear SVM on top of the graph representations for classification. The classification was performed to assess the discriminative power of the learned graph-level representations.

## RESULTS AND DISCUSSION

In this section, we conducted extensive experiments to evaluate the performance of our model. Based on the different PPI networks for the CG identification task, our model outperformed several existing state-of-the-art networks and multi-omic aggregation methods. To evaluate the generalization capability of our model, we further tested our model on other independent node classification and graph classification tasks, including EG identification, HD gene identification and disease subnetwork identification tasks. Moreover, to provide a more intuitive interpretation of our model, we utilized GNNExplainer [45] to analyze the contribution of the edges and features during model training and correlated gradient methods [46] to analyze the contribution of each node.

### Implementation details

The model was implemented using the Pytorch framework. All the experiments were conducted on one Nvidia A100 GPU. The input graphs were the multi-omic featured PPI networks irrespective of the different downstream tasks. In the SMG reconstruction phase, the mask ratio was set as 0.5, indicating that 50% nodes of each graph were replaced by the MASK and needed the reconstruction. We trained our network using the Adam optimizer for 300 epochs for the first two tasks and 100 epochs for the final HD gene identification task, respectively. The learning rate and weight decay were initialized as 0.1 and 0, respectively. In the task-specific fine-tuning phase, we adopted the Adam optimizer and set the learning rate and weight decay as 0.01 and 0.001, respectively. To ensure a fair comparison, we followed the splitting strategy of EMOGI [20] for model training and testing. The training and test data were randomly split independently. Detailed data distribution in terms of the positive, negative and unlabelled samples is provided in the Supplementary Material. Five-fold cross-validation test was then performed to evaluate the network performance. We used the area under the precision-recall curve (AUPRC) as the primary evaluation metric. The GNN architecture in our model was constructed by three hidden layers (except the input layer), and the dimension of each hidden layer was 256. Each layer is followed by a layer normalization function and a Rectified Linear Unit (ReLU) activation function. At the supervised fine-tuning stage, the early stopping strategy was applied to monitor the performance of the model for each epoch on a held-out validation set during the model training. The validation set was extracted from the training set to ensure the independence from the test set; we utilized the ratio of 4:1 for splitting the final training and validation sets. At the fine-tuning stage, we chose the best-performing model as the final model to evaluate the model performance on the test set.

### Performance on CG identification

**Figure 2:**
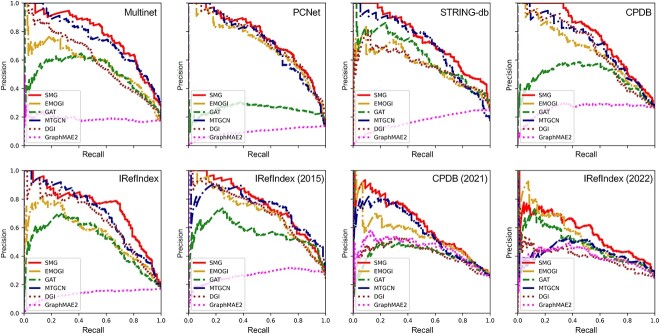
Performance comparison of SMG with other state-of-the-art methods on CG identification task in terms of PRCs across eight PPI networks. Different methods are indicated by different colours.

We compared our proposed SMG method with other state-of-the-art methods on the CG identification task across eight different PPI networks. The experimental results and precision-recall curves (PRCs) were presented in Table 1 and Figure 2, respectively. EMOGI [20] is a representative method for identifying CGs based on semi-supervised node classification. However, a caveat of this approach lied in neglecting the valuable information in the unsampled data. MTGCN [22] attempted to leverage the graph knowledge by multitask learning. More specifically, they introduced the link prediction subtask to embed more topological information in the representations. In another recent work, DGI [38] utilized the graph attention network to capture more global information in the PPI networks. GAT [42] is another well-known graph attention network, which has been widely applied to address graph-based feature extraction tasks. As shown in Table 1, the convolution-based EMOGI method [20] performed better than the attention-based GAT method [42], indicating that CGs tend to be closely related to their neighbour nodes, whereas the global information contributed less to their identification. Meanwhile, both SMG and MTGCN [22] achieved better performance than other methods, implying that incorporating more relevant knowledge and useful constraints could help improve the model performance. Additionally, we also compared our method with the latest graph self-supervised model, GraphMAE2 [62]. We can see that the performance of GraphMAE2 was relatively inferior, which might be attributed to the negative transfer phenomenon in graph self-supervised training. That is, the complex and difficult pretraining may exert negative influence on the downstream tasks. Benefiting from the SMG’s capability to simultaneously capture the holistic topological information of the graph structure and the interaction relationships amongst the local nodes’ features, SMG achieved the best performance in terms of the AUPRC values across all eight PPI networks, with an AUPRC performance improvement of overall average 7.4% compared with the second-best model on each data set. Figure 2 shows an example of the PRCs of all methods across eight different PPI networks in the last-fold experiments. The proposed SMG method, indicated by the red curves in Figure 2, consistently achieved the best performance, highlighting its excellent predictive performance and generalization capability.

**Table 1:** Performance comparison of SMG with other state-of-the-art methods on CG identification task in terms of AUPRC in the min/median/max format.

Method Networks	DGI [38]	GAT [42]	EMOGI [20]	MTGCN [22]	GraphMAE2 [63]	SMG
**CPDB**	0.7614/0.7454/0.7412	0.6466/0.5810/0.4890	0.7544/0.6963/0.6568	0.8036/0.7861/0.7370	0.5485/0.4750/0.4050	**0.8148/0.8008/0.7588**
**IRefIndex**	0.6452/0.6423/0.6392	0.5433/0.4822/0.3521	0.5825/0.5557/0.4536	0.7568/0.7150/0.6720	0.3563/0.2978/0.2899	**0.7627/0.7100/0.6682**
**PCNeT**	0.7481/0.7428/0.7394	0.4520/0.2709/0.2168	0.7543/0.6991/0.6797	0.7922/0.7717/0.7502	0.2562/0.2393/0.2310	**0.8041/0.7670/0.7318**
**IRefIndex (2015)**	0.7027/0.6993/0.6933	0.5707/0.5347/0.5197	0.7882/0.7303/0.7091	0.8189/0.7810/0.7652	0.5352/0.4910/0.4623	**0.8107/0.7823/0.7483**
**STRING-db**	0.6765/0.6366/0.5255	0.7329/0.6985/0.6462	0.7285/0.6678/0.6326	0.7985/0.7607/0.6751	0.5962/0.5913/0.5038	**0.7929/0.7525/0.7281**
**Multinet**	0.6818/0.6695/0.6606	0.5122/0.4613/0.4110	0.7025/0.6139/0.6001	0.7956/0.7667/0.7459	0.4943/0.4564/0.4364	**0.8209/0.7653/0.7500**
**CPDB (2021)**	0.4764/0.4720/0.4712	0.3694/0.3516/0.3079	0.5475/0.5262/0.5045	0.5904/0.5719/0.5475	0.4801/0.4112/0.4078	**0.6398/0.5871/0.5395**
**IRefIndex (2022)**	0.4232/0.3819/0.3598	0.3811/0.3781/0.3594	0.4925/0.4449/0.3968	0.4849/0.4312/0.4203	0.4089/0.3819/0.3773	**0.5972/0.5295/0.4977**

The best performance was indicated in bold.

**Figure 3:**
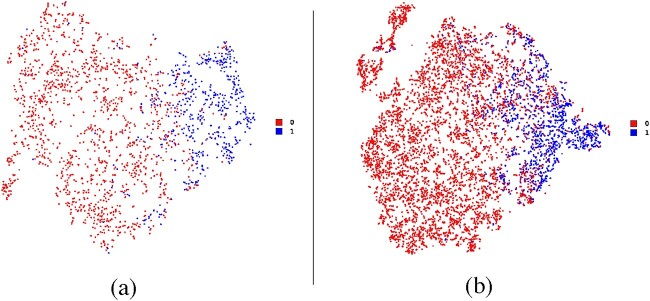
Visualization of the feature distributions of CGs (**A**) and EGs (**B**) through t-SNE. Category 0 and 1 represent the negative and positive samples, respectively.

In addition to the quantitative performance comparison, we also qualitatively visualized the feature distribution of CGs identified by SMG using t-SNE [64] in Figure 3A, where the positive and negative genes are, respectively, coloured in blue and red. The feature distributions in the feature space clearly indicate that the positive and negative genes exhibit distinct patterns, highlighting the remarkable feature extraction capability of the proposed SMG for discriminating the positives from negatives.

### Performance on EG identification

Except for the CG identification task, the multi-omic featured PPI networks can also be applied to the EG identification task. Similar to CG identification, EG identification is also faced with the insufficiency issue of labelled data, making its identification extremely challenging. Here, we utilized the same multi-omic featured graphs as those of the CG identification task and provided the corresponding performance results in Table 2. As can be seen, SMG clearly outperformed other methods across the eight different PPI networks and achieved an overall average performance improvement of 8.7% in terms of AUPRC values compared with the second best model across the PPI data sets. These results illustrate the superiority of SMG on feature representation and generalization. Moreover, the feature distributions of the EGs can be visualized in Figure 3B. Apparently, the positive and negative samples exhibited distinct distribution patterns in the feature space, demonstrating that our model can capture label-related semantic information to a significant extent.

**Table 2:** Performance comparison of SMG with other state-of-the-art methods on EG identification task in terms of AUPRC in the min/median/max format

Method Networks	DGI [38]	GAT [42]	EMOGI [20]	MTGCN [22]	GraphMAE2 [63]	SMG
**CPDB**	0.7267/0.7233/0.7207	0.6568/0.6429/0.5822	0.7717/0.7550/0.7278	0.7434/0.7178/0.6895	0.5441/0.5161/0.5009	**0.8444/0.8137/0.7735**
**IRefIndex**	0.6735/0.6707/0.6659	0.5788/0.5632/0.5556	0.6981/0.6868/0.6736	0.6810/0.6618/0.6312	0.5283/0.4955/0.4743	**0.8047/0.7836/0.7577**
**PCNeT**	0.7907/0.7866/0.7861	0.6815/0.5988/0.5411	0.8073/0.7863/0.7757	0.7604/0.7548/0.6813	0.5483/0.5279/0.5215	**0.8466/0.8273/0.8027**
**IRefIndex (2015)**	0.7227/0.7195/0.7121	0.5716/0.5612/0.5360	0.7308/0.7014/0.6770	0.7326/0.7032/0.6705	0.5137/0.4955/0.4871	**0.7930/0.7783/0.7536**
**STRING-db**	0.7856/0.7740/0.7615	0.7572/0.6835/0.6795	0.8029/0.7747/0.7648	0.7316/0.7060/0.6912	0.6503/0.6289/0.5775	**0.8623/0.8591/0.8262**
**Multinet**	0.6364/0.6296/0.6195	0.5123/0.4982/0.4199	0.6690/0.6441/0.5846	0.6463/0.6130/0.5693	0.3962/0.3701/0.3358	**0.7759/0.7087/0.7029**
**CPDB (2021)**	0.6678/0.6652/0.6595	0.5454/0.5386/0.4679	0.7341/0.6802/0.6662	0.6464/0.6243/0.6040	0.6622/0.6187/0.5976	**0.7642/0.7309/0.7096**
**IRefIndex (2022)**	0.5740/0.5695/0.5655	0.6075/0.5738/0.4724	0.6541/0.6422/0.6239	0.5659/0.5508/0.5234	0.5475/0.5422/0.5129	**0.7180/0.7019/0.6753**

### Performance on HD gene identification

Compared with fully supervised methods, the semi-supervised methods only have a small portion of labelled samples, which could dramatically limit the model performance. Here, we aimed to assess the limitations of the semi-supervised fine-tuning strategy on the HD gene identification task. According to NCG [35], there are currently 95 HD genes and accordingly, we sampled those that can be revealed on the PPI networks. Particularly, we randomly sampled the same number of unlabelled samples as the negative samples instead of using the previous 1:3 ratio, considering that the number of the positive samples in this task was relatively small and the oversampling of the negatives would potentially impact the model training. Then, we chose the splitting ratio of 4:1 between the training and test data to conduct the model training and test. Therefore, a majority of the nodes on the graph were unlabelled, containing only a small portion of the labelled data (around 1%). The results are provided in Table 3. As can be seen, even under extreme lacking labelled data conditions, our proposed SMG achieved remarkable performance, with a maximum AUPRC value of 0.9639. These results highlight the excellent capability of SMG in learning feature representations under semi-supervised conditions where limited amount of labelled data are available.

**Table 3:** Performance comparison of SMG with other state-of-the-art methods on health driver gene identification task in terms of AUPRC in the min/median/max format

Method Networks	DGI [38]	GAT [42]	EMOGI [20]	MTGCN [22]	GraphMAE2 [63]	SMG
**CPDB**	0.8062/0.7882/0.7837	0.7797/0.5473/0.5155	0.7752/0.7373/0.5966	0.9020/0.8182/0.7478	0.8465/0.7173/0.5939	**0.9124/0.8600/0.7248**
**IRefIndex**	0.8626/0.8511/0.8459	0.7374/0.6133/0.4730	0.8213/0.6628/0.5675	0.9003/0.8916/0.7266	0.7466/0.7277/0.5739	**0.9256/0.8785/0.7942**
**PCNeT**	0.8640/0.8432/0.8312	0.7174/0.6019/0.5128	0.8394/0.7524/0.5785	0.9352/0.8331/0.7777	0.9064/0.7550/0.6743	**0.9379/0.8609/0.8269**
**IRefIndex (2015)**	0.8020/0.7725/0.6608	0.7373/0.6370/0.3988	0.8372/0.6486/0.5664	0.8897/0.8302/0.5125	0.8476/0.7380/0.5634	**0.8482/0.7620/0.7261**
**STRING-db**	0.8037/0.7744/0.7424	0.9368/0.6496/0.5114	0.8737/0.6776/0.6680	0.9040/0.8876/0.7400	0.8352/0.7508/0.5866	**0.9639/0.8899/0.8099**
**Multinet**	0.5350/0.5159/0.5050	0.7793/0.6456/0.6203	0.7997/0.6843/0.5883	0.9063/0.8488/0.6989	0.8184/0.7466/0.6677	**0.9451/0.8596/0.6282**
**CPDB (2021)**	0.5656/0.5493/0.5023	0.7134/0.6045/0.4864	0.7862/0.6550/0.5715	0.8855/0.8505/0.7833	0.8621/0.7497/0.5214	**0.9183/0.8503/0.6523**
**IRefIndex (2022)**	0.6172/0.5582/0.5235	0.7478/0.6463/0.5331	0.8117/0.7600/0.7152	0.8251/0.7254/0.5841	0.6882/0.5835/0.5606	**0.9073/0.8249/0.7533**

### Generalization evaluation

**Figure 4:**
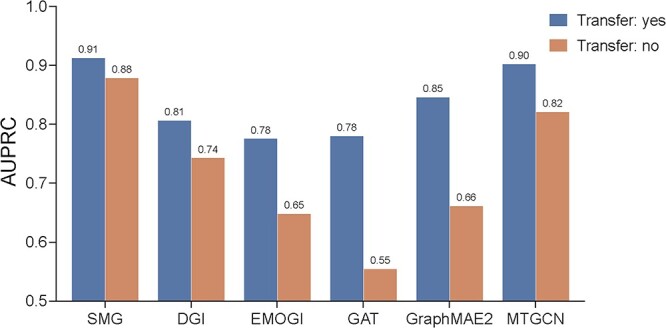
The experiment results of the transfer testing.

In this subsection, we examined the models’ generalization capability by designing an independent transfer testing task. Specifically, we tested the model performance on two settings to assess the learned features’ generalization for different downstream tasks. Under the transfer setting, the encoder undergoes fine-tuning using the HD genes, whereas under the without-transfer setting, the encoder is fine-tuned using the CG labels. Both models were tested on the HD gene data set. The best performance results during the five-fold cross-validation are illustrated in Figure 4. Here, we used the CPDB PPI network for graph construction. We can see that for both SMG and other compared methods, the models with transfer performed better than those without transfer. Nevertheless, compared with the corresponding model with transfer, the SMG model without transfer had a smaller performance decrease in contrast to other methods. These results illustrate that our proposed SMG method has a powerful representation learning capability by combining the GNN encoder, thereby being able to achieve outstanding performance in various downstream tasks. Meanwhile, most deep learning models are. In addition, by generating the discriminative feature representations, SMG can effectively alleviate the issues of having inferior scalability and propensity for overfitting.

### Performance on the disease subnetwork identification task

**Figure 5:**
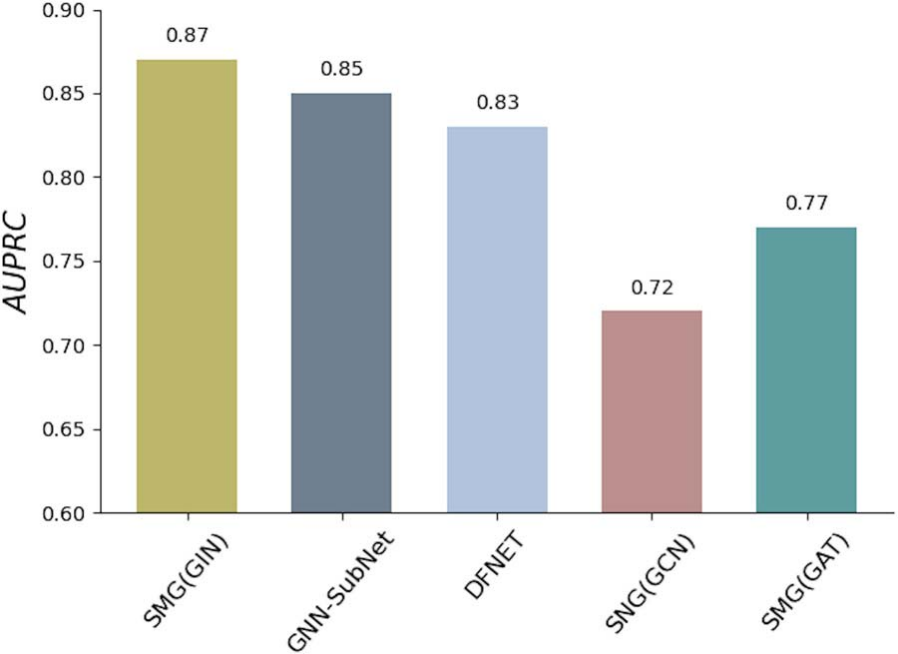
The experiment results of the graph classification task on disease subnetwork detection.

The above experiments have demonstrated SMG’s node-level feature representation capability. In this subsection, we further tested its generalization on graph-level tasks (i.e. the disease subnetwork identification task). Similar to GraphSubNet, we first employed the GNN encoder to extract features and subsequently used an average readout function to transform the graph into a global representation. Then, we input the extracted features into a linear SVM to obtain the label assignment. As shown in Figure 5, SMG achieved the highest AUPRC of 0.87, clearly outperforming all other methods. In summary, the remarkable performance of SMG on graph-level tasks highlights its ability to learn useful representative features through self-supervised pre-training.

### Derived potential CGs through SMG

In this subsection, we discussed the computationally identified CGs by the proposed SMG method. Specifically, we generated the confidence scores of all the unknown genes by applying the sigmoid function to the output from the task-specific layer and analyzed the top-ranked nodes [i.e. dystonin (DST), KMT2B and SETD2] below.

#### Dystonin

Aberrant expression of DST has been observed in many cancer types. For example, Chang *et al*. [47] and Komatsu *et al*. [48] reported that the expression of DST is different in breast normal and tumour tissues, and the expression level of the DST mRNA tends to be lower in breast tumour tissue. Furthermore, the expression of the DST mRNA has a close correlation with recurrence-free survival in luminal A subtype cancer patients [49]. Apart from the differential expression analysis, the molecular mechanism of DST on breast cancer was also reported [50]. It was found that the missing of DST could promote the progression of breast cancer and DST could prevent tumour growth and invasion [50]. Thereby, the downregulation of DST has a significant correlation with cancer, which can be regarded as a potential candidate of CGs.

#### KMT2B

Alteration of KMT2B has been widely observed in 5.1% of cancers [51]. For instance, Ghanbari *et al*. [52] analyzed the cancer tissue collected from 43 female patients and utilized the RNA extraction, cDNA synthesis and quantitative PCR to evaluate the expression level of the KMT2B gene. They revealed that the KMT2B expression levels were significantly downregulated in breast cancer tissues compared with marginal tissues. Alternatively, it has also been reported that the non-synonymous mutations in human cancers, including Endometrial, large intestine, lung, glioma and liver carcinomas contain 72% of identified mutations in the coding region of KMT2B [53]. These findings provide evidence suggesting that KMT2B could be a potential candidate of cancer-related genes.

#### SETD2

Alteration of SETD2 has been also widely observed in 3.57% of all cancers [50]. Notably, lung adenocarcinoma, colon adenocarcinoma, clear cell renal cell carcinoma, breast invasive ductal carcinoma and endometrial endometrioid adenocarcinoma exhibit the highest prevalence of alterations [51]. Lu *et al*. [54] investigated the role of SETD2 somatic mutation in the pan-cancer cohort and revealed that the SETD2 mutant patients have higher tumour mutation burden and microsatellite instability compared with SETD2 nonmutant patients. Meanwhile, Newbold *et al*. [55] analyzed the SETD2 expression at the mRNA and protein levels in solid cancer and suggested that SETD2 can possibly act as a tumour suppressor, thereby highlighting the cancer-related gene characteristics of SETD2.

### Case study using the GNNExplainer and integrated gradient

In this subsection, we conducted case studies to better understand the prediction process and interpret the potential knowledge learned by the network. Here, we took the well-known PIK3CA as an example and utilized GNNExplainer [45] and Integrated Gradient [46] for interpretation. GNNExplainer [45] can identify a compact subgraph structure and a small subset of node features that contribute to the GNN’s prediction, whereas the Integrated Gradient [46] is able to introduce two fundamental axioms (i.e. sensitivity and implementation invariance) to enable model interpretation.

For each node in CG identification, we can find a target subgraph that reveals the interactions with its neighbour nodes. The GAB1-PIK3CA is the most important gene pair. The interaction between the GAB1 and PIK3CA reflects the Gab1/PI3K/Akt transduction pathway. PI3K-Akt [56–58] is one of the most deregulated pathways in cancer and has been implicated in various types of cancer. Meanwhile, GAB1 is a scaffold protein involved in numerous interactions propagating signalling by growth factor and cytokine receptors. Kiyatkin *et al*. [59] reported that the expression of Gab1 has a highly close relationship with the PI3K/Akt pathway, and the interference of Gab1 expression via siRNA can decrease the PI3K/Akt pathway activity in the intrahepatic cholangiocarcinoma cell line RBE. This indicates the molecular mechanism for cancer of GAB-PIK3CA through the Gab1/PI3K/Akt transduction pathway. The aforementioned molecular mechanism validation of the attribution result of our model prediction based on the GNNExplainer illustrates the reliability of our model’s prediction. Moreover, because of its trustworthy interpretation results, researchers can apply the SMG method to propose and test underlying molecular mechanisms associated with the cancer-related genes in the future.

The attribution results of GNN were mainly concerned with the topological structure given by the weighted subgraph and feature. Therefore, for the node-level interpretation, we chose the advanced Integrated Gradient for achieving the model interpretation. Compared with Layerwise Relevance Propagation proposed in EMOGI, the Integrated Gradient method satisfies the implementation invariance axiom, i.e. the attribution outcome of the two different functionally equivalent networks will be invariant, which is more suitable for the deep learning model. For the neural network and input feature, we only need to set a baseline input to measure the input change with the initial state input. In this way, the integrated value would reflect the inputs’ attribution value.

We further analyzed the node attribution ranking for the PI3KCA prediction result. Similarly, we only discussed the top-2 ranking nodes (i.e., AFDN and SMURF2). In terms of SMURF2, David *et al*. [60] demonstrated that silencing of Smurf2 could downregulate the proliferation of breast cancer cells by modulating the PI3K-PTEN-AKT-FoxO3a pathway, which means that Smurf2 is a vital component for the PI3K pathway as cancer-related gene. In the case of AFDN, Goudreault *et al*. [61] reported that knockout of AFDN in MCF7 epithelial cells disrupts MAPK and PI3K activation and inhibits cell motility in a growth factor-dependent manner, indirectly indicating the correlation between AFDN and PI3K. Such correlation might further influence the PI3K-AKT pathway for cancer occurrence. Moreover, Goudreault *et al*. [61] observed the nuclear localization of Afadin in clinical breast cancer specimens, which indicates the regulation of Afadin by the PI3K-AKT pathway has pathophysiological significance [61]. Overall, the molecular mechanistic studies provide powerful support for our model’s prediction of the P13K as a cancer driver gene.

**Figure 6:**
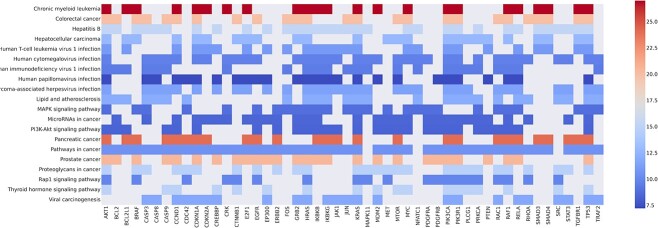
Selected heatmap of gene set enrichment analysis based on the top-ranked genes in the attribution results on the KEGG pathway. The colour of each slot represents the odds ratio of the enrichment analysis.

**Figure 7:**
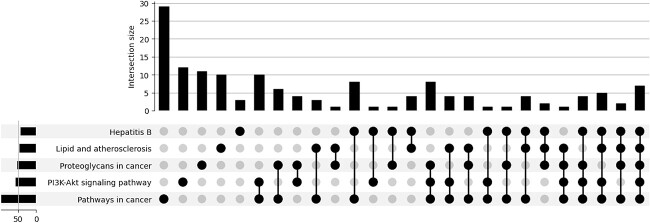
Selected UpSet plot of gene set enrichment analysis based on the top-ranked genes in the attribution results on the KEGG pathway. The size of the intersection is defined by the number of shared genes.

**Figure 8:**
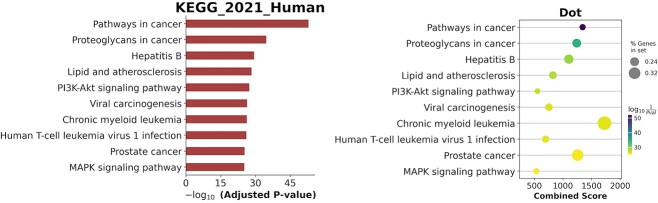
Visualization of gene set enrichment analysis based on the top-ranked genes in the attribution results on the KEGG pathway. Pathways are ranked by the combined scores.

In addition to the independent analysis of each top-ranked gene, we also used the top-ranked gene set to perform pathway enrichment analysis, in an attempt to validate the plausibility of the Integrated Gradient attribution result. To accomplish this, we employed the Enrichr API [65] with the KEGG-2021-human data set [66], by performing the hypergeometric hypothesis test for the pathway enrichment analysis. Figure 6 displays genes that occurred in more than seven pathways and shows that the overlapping pathways are all related to cancer. Figure 7 shows that indeed the combination of cancer-related pathways dominated the enrichment result of the top-ranked gene sets. Figure 8 lists the top-ranked pathways with a false discovery rate control level set at 0.25. It can be observed that the top-ranked pathways are predominantly related to cancer pathways. Altogether, our SMG model can provide plausible biological interpretability of the identified CGs.

In summary, we conducted both node- and edge-level assessment of the trained SMG model, providing supportive evidence for its superior performance and reliability. We envision that SMG will serve as a useful approach for predicting novel potential CGs and helping elucidate the molecular-level mechanisms from the PPI network data.

## CONCLUSIONS

In this study, we have developed a novel SMG learning framework termed SMG for identifying CGs based on the multi-omic featured PPI networks. Benchmarking experiments showed that SMG could achieve competitive performance compared with previous benchmarking methods on eight PPI networks and additional subtasks, including EG, HD gene identification and disease subgraph identification. The results demonstrated the excellent generalization and scalability of the proposed SMG framework. To improve the model interpretability from the biological perspective, we leveraged two deep neural network-based interpretation tools to explain its prediction process. We envision that the proposed SMG method can be explored as a useful tool to accelerate data-driven discovery of cancer-related genes and help characterize their molecular mechanisms based on the PPI network data.

**Key Points**

- Identifying cancer genes (CGs) associated with the initiation and progression of cancer is important for elucidation of molecular-level mechanism in cancer research.
- We propose a novel self-supervised masked graph (SMG) learning approach, termed SMG, for identifying CGs from multi-omic featured PPI networks.
- Benchmarking tests on three node-level tasks and one graph-level task across eight PPI networks demonstrate that SMG achieves outstanding performance compared with other state-of-the-art methods.

## Supplementary Material

supplementary_material_bbad406

## Data Availability

The source codes and data sets curated in this study are available on GitHub at https://github.com/C0nc/SMG.

## Author Biographies

**Yan Cui** is currently a master student at the Bioinformatics Center, Institute for Chemical Research, Kyoto University, Japan. His research interests include machine learning and computational biology.

**Zhikang Wang** is currently a PhD student in the Department of Biochemistry and Molecular Biology and Biomedicine Discovery Institute, Monash University, Australia. His interests are computational pathology, bioinformatics, pattern recognition and deep learning.

**Xiaoyu Wang** received her MS degree in information technology from The University of Melbourne in 2020. She is currently a PhD student in the Department of Biochemistry and Molecular Biology and Biomedicine Discovery Institute, Monash University. Her research interests include bioinformatics, computational biomedicine and machine learning.

**Yiwen Zhang** received her MS degree in business analytics from Monash University in 2022. She is currently a PhD student at the School of Public Health and Preventive Medicine, Monash University, and a member of the Climate, Air Quality Research group. Her research interests include planetary and environmental health, public health and epidemiology, and biostatistics.

**Ying Zhang** is currently a PhD candidate in the School of Computer Science and Engineering at Nanjing University of Science and Technology, China. Her research interests include bioinformatics, machine learning and pattern recognition.

**Tong Pan** is currently a PhD candidate in the Department of Biochemistry and Molecular Biology and Biomedicine Discovery Institute, Monash University, Australia. Her research interests include bioinformatics, machine learning and protein function recognition.

**Zhe Zhang** received his master’s degree in informatics from Kyoto University in 2019. He is currently working as a researcher at UniDT Co., Ltd. His research interests include combinatorial optimization, integer linear programming and reinforcement learning.

**Shanshan Li** is an associate professor of global environmental health and biostatistics and deputy head of the Monash Climate, Air Quality Research (CARE) Unit. Her research interests include planetary and environmental health, child health, public health and epidemiology, and biostatistics.

**Yuming Guo** is a professor of global environmental health and biostatistics and head of the Monash Climate, Air Quality Research (CARE) Unit. His research focuses on environmental epidemiology, biostatistics, global environmental change, air pollution, climate change, urban design, residential environment, remote sensing modelling and infectious disease modelling.

**Tatsuya Akutsu** received his DEng degree in information engineering in 1989 from the University of Tokyo, Japan. Since 2001, he has been a professor in the Bioinformatics Center, Institute for Chemical Research, Kyoto University, Japan. His research interests include bioinformatics and discrete algorithms.

**Jiangning Song** is currently an associate professor and group leader in the Monash Biomedicine Discovery Institute, Monash University. He is also affiliated with the Monash Data Futures Institute, Monash University. His research interests include bioinformatics, computational biology, machine learning, data mining and pattern recognition.
