# Supplementary material for: SMG: self-supervised masked graph learning for cancer gene identification
Source: Brief Bioinform. 2023 Nov 8;24(6):bbad406. doi: 10.1093/bib/bbad406 (PMC10639095; doi:10.1093/bib/bbad406)
Supplement: supplementary_material_bbad406 [file supplementary_material_bbad406.docx]

**Supplementary Material**

**Fig. S1.** Pie charts illustrate the positive, negative, and unlabeled sample percentage in cancer gene, essential gene, and healthy driver gene identification tasks across eight PPI networks.

**Table S1.** Statistical summary of samples across the three tasks on the eight PPI networks in the (positive/negative/unlabeled) format.

| **Network**  **Task** | **CPDB** | **IRefIndex** | **PCNET** | **IRefIndex (2015)** |
| --- | --- | --- | --- | --- |
| **1** | 796/ 2,187/ 10,644 | 836/ 4,056/ 12,121 | 859/ 5,483/ 13,439 | 785/ 1,973/ 9,371 |
| **2** | 1,830/ 6,861/ 4,936 | 1,926/ 9,455/ 5,632 | 2,024/ 11,601/ 6,156 | 1,833/ 5,746/ 4,550 |
| **3** | 90/ 90/ 13,447 | 92/ 92/ 16,829 | 95/ 95/ 19,591 | 84/ 84/ 11,961 |
| **Network**  **Task** | STRING-db | Mutlinet | CPDB (2021) | IRefIndex (2022) |
| **1** | 783/ 2,415/ 9,981 | 790/ 3,709/ 9,899 | 786/ 2,358/ 11,036 | 770/ 2,310/ 14,097 |
| **2** | 1,861/ 6,697/ 4,621 | 1,817/ 7,821/ 4,760 | 1,799/ 5,397/ 6,984 | 1,747/ 5,241/ 10,189 |
| **3** | 87/ 87/ 13,005 | 84/ 84/ 14,230 | 92/ 92/ 13,996 | 92/ 92/ 16,993 |

**Data distribution of the three tasks across eight PPI networks**

From **Fig. S1** and **Table S1**, we can see the percentage and number of positive, negative, and unlabelled samples on the three node-level tasks across eight PPI networks. The numbers of positive samples are much less than those of the negative and unlabelled ones. Especially in terms of the health driver gene identification task, the positive samples are less than 1%. The model performance on this specific task can serve to illustrate the robustness and generalization capabilities under extreme data imbalance conditions.

**
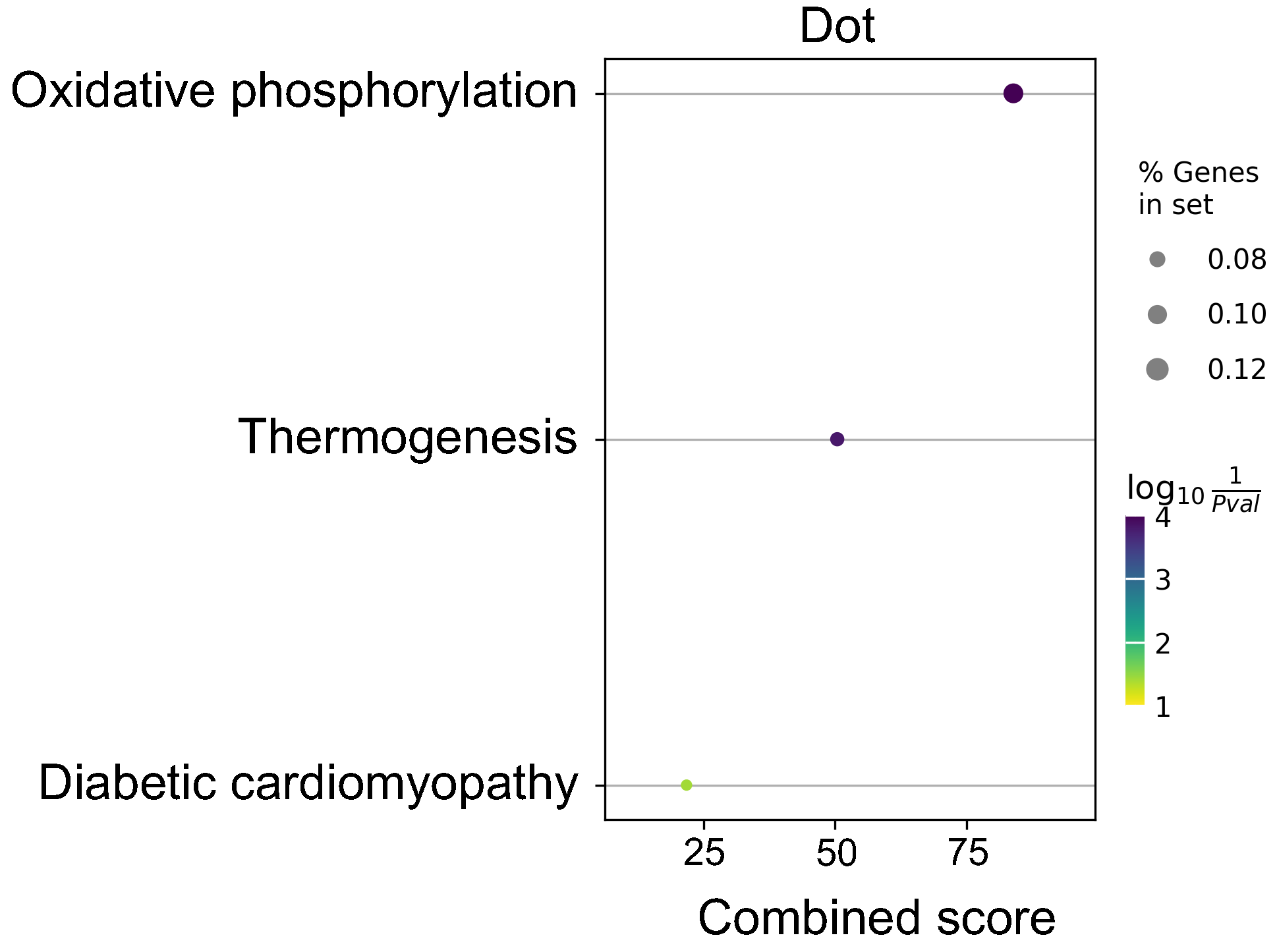
**

**Fig. S2.** Illustration of the bottom-ranked gene sets, which are only related to three significant enriched pathways, i.e. oxidative phosphorylation, thermogenesis and diabetic cardiomyopathy, all of which are not related to the cancer.
